# Supplementary material for: Transcriptomic Insights into Mechanisms of Early Seed Maturation in the Garden Pea (Pisum sativum L.)
Source: Cells. 2020 Mar 23;9(3):779. doi: 10.3390/cells9030779 (PMC7140803; doi:10.3390/cells9030779)

Zhewan−1,25(1)

Global Summary

%DE = 0.29

genes with fdr < 0.2 = 174 (74 + / 100 −)

genes with fdr < 0.1 = 163 (68 + / 95 −)

genes with fdr < 0.05 = 163 (68 + / 95 −)

genes with fdr < 0.01 = 140 (60 + / 80 −)

genes in genesets = 0

<FC> = 0

<t−score> = 0

<p−value> = 0

<fdr> = 0.71

Portrait

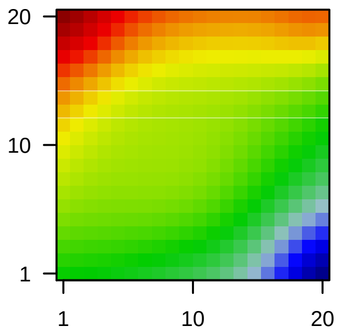

Top 100 DE genes

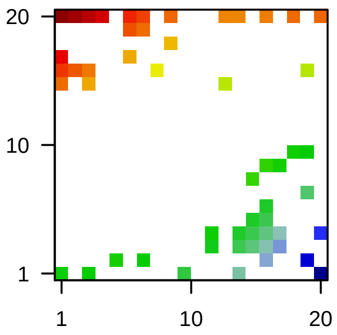

Global Genelist

Rank	ID	log(FC)	p−value	fdr	Metagene	Description
1	TRINITY_DN2	−0.17	2e−16	1e−15	14 x 3	
2	TRINITY_DN1	−9.53	2e−16	1e−15	20 x 1	
3	TRINITY_DN1	3.01	2e−16	1e−15	20 x 20	
4	TRINITY_DN5	0.51	2e−16	1e−15	2 x 20	
5	TRINITY_DN1	0.2	2e−16	1e−15	7 x 20	
6	TRINITY_DN1	−0.2	2e−16	1e−15	10 x 1	
7	TRINITY_DN1	0.17	2e−16	1e−15	7 x 19	
8	TRINITY_DN3	0.26	2e−16	1e−15	1 x 17	
9	TRINITY_DN5	−0.15	2e−16	1e−15	12 x 3	
10	TRINITY_DN2	−3.11	2e−16	1e−15	20 x 1	
11	TRINITY_DN2	−0.91	2e−16	1e−15	20 x 1	
12	TRINITY_DN3	0.14	2e−16	1e−15	9 x 18	
13	TRINITY_DN3	0.2	2e−16	1e−15	7 x 20	
14	TRINITY_DN3	−0.18	2e−16	1e−15	19 x 10	
15	TRINITY_DN5	0.24	2e−16	1e−15	1 x 1	
16	TRINITY_DN1	−0.17	2e−16	1e−15	14 x 3	
17	TRINITY_DN7	−0.14	2e−16	1e−15	15 x 5	
18	TRINITY_DN4	−0.22	2e−16	1e−15	15 x 3	
19	TRINITY_DN4	−0.7	2e−16	1e−15	1 x 1	
20	TRINITY_DN4	0.16	2e−16	1e−15	18 x 20	

p−values

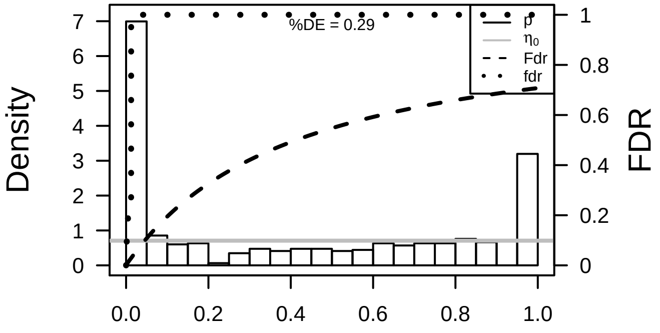

Supplement: Supplementary file 1 [file cells-09-00779-s001.zip › Supplementary materials/FileS3.zip!/FileS2/Hormones - Results/Summary Sheets - Samples/Zhewan.1.25.1..pdf]
